# Influence of pre-analytical sample preparation on drug concentration measurements in peritoneal tissue: an *ex-vivo* study

**DOI:** 10.1515/pp-2020-0151

**Published:** 2021-07-28

**Authors:** Arianna Castagna, Iaroslav Sautkin, Frank-Jürgen Weinreich, Hannah Heejung Lee, Alfred Königsrainer, Marc André Reymond, Giorgi Nadiradze

**Affiliations:** Department of General, Visceral and Transplant Surgery, University of Tübingen, Tübingen, Germany; National Center for Pleura and Peritoneum, Comprehensive Cancer Center Tübingen – Stuttgart (CCC-TS), Tübingen, Germany

**Keywords:** cisplatin, doxorubicin, enhanced inverted Bovine Urinary Bladder (eIBUB), pre-analytical studies, sample geometry, tissue drug concentration

## Abstract

**Objectives:**

Biopsy morphology (surface/depth ratio) and sample processing might affect pharmacological measurements in peritoneal tissue.

**Methods:**

This is an *ex-vivo* study on inverted bovine urinary bladders (IBUB). We compared cisplatin (CIS) and doxorubicin (DOX) concentration in 81 standardized transmural punch biopsies of different diameters (6 and 12 mm). Then, we assessed the effect of dabbing the peritoneal surface before analysis. After automatized tissue homogenization with ceramic beads followed by lyophilisation, DOX concentration was quantified by high-performance liquid chromatography (HPLC), CIS concentration by atomic absorption spectroscopy. Experiments were performed in triplicate; the analysis was blinded to the sample origin. Comparisons were performed using non-parametric tests.

**Results:**

Concentrations are given in mean (CI 5–95%). Results were reproducible between experiments (for CIS p=0.783, for DOX p=0.235) and between different localizations within the IBUB (for CIS p=0.032, for DOX p=0.663). Biopsy diameter had an influence on CIS tissue concentration (6 mm biopsies: 23.2 (20.3–26.1), vs. 12 mm biopsies: 8.1 (7.2–9.2) ng/mg, p<0.001) but not on DOX: (0.46, 0.29–0.62) vs. 0.43 (0.33–0.54) ng/mg respectively, p=0.248). Dabbing the peritoneal surface reduced DOX tissue concentration (dry biopsies: 0.28 (0.12–0.43) vs. wet biopsies: 0.64 (0.35–0.93) ng/mg, p=0.025) but not CIS (23.5 (19.0–28.0) vs. 22.9 (18.9–26.9) ng/mg, respectively, p=0.735).

**Conclusions:**

Measurements of drug concentration in peritoneal tissue can be influenced by the biopsy’s surface/depth ratio and after drying the biopsy’s surface**.** This influence can reach a factor three, depending on the drug tested**.** The biopsy technique and the pre-analytical sample preparation should be standardized to ensure reliable pharmacological measurements in peritoneal tissue.

## Introduction

Studies on peritoneal metastasis (PM) and its treatment aim to improve the life expectancy and quality of affected patients. New therapies such as cytoreductive surgery, targeted agents, and locoregional chemotherapy have been shown to prolong survival [1, 2]. The importance of complete macroscopic cytoreductive surgery (CRS) has been increasingly recognized because of the limited drug availability in the tumor nodules [3], [4], [5]. A randomized phase 3 trial showed significant benefit in recurrence-free and overall survival when Hyperthermic IntraPeritoneal Chemotherapy (HIPEC) was added to interval CRS in patients who were not eligible for primary surgery because of the extent of their disease [6]. However, whereas the role of optimal cytoreductive surgery was confirmed, a survival benefit of additional HIPEC could not be demonstrated in colorectal cancer [7]. Thus, there is a need for optimizing drug delivery techniques for intraperitoneal chemotherapy.

An innovative drug delivery technique, Pressurized IntraPeritoneal Chemotherapy (PIPAC) has been shown to improve drug availability in peritoneal tissue. By applying an artificial hydrostatic pressure, PIPAC increases the depth of tissue penetration compared to conventional intraperitoneal chemotherapy with liquids [3, 8]. In contrast to HIPEC, PIPAC can be repeated, and its therapeutic effect can be assessed objectively by comparing tumor biopsies taken at each cycle, for example, by using a dedicated tumor regression grading [9]. The drug uptake increases after every application in the tumor and in the ascites [3, 5, 10].

Representative models of the peritoneal tissue are required for optimizing drug distribution. Schnelle et al. [11] introduced a simple *ex-vivo* model of the peritoneal surface, enabling pharmacological studies. This model has been further developed to allow real-time measurement and differentiates between tissue uptake from an aerosol vs. a liquid [12]. In this *ex-vivo* model, multiple tissue biopsies are taken for pharmacological measures; however, there is no data on pre-analytical steps and their influence on drug concentration values. For example, it can be hypothesized that the drug concentration will be maximal at the surface of a biopsy and diminish with distance from this surface. Thus, the geometry of the biopsy, defined as the ratio between the exposed surface and the depth, might influence pharmacological results. Another possible bias would be a chemotherapy film remaining at the surface of the biopsy, with no chemotherapy taken up into the tissue. In such case, tissue drug concentration would be artificially high.

This methodological study focuses on the influence of pre-analytical steps on biopsy measurements of drug concentration in peritoneal tissue. Specifically, we examined the tissue concentration of two different chemotherapeutic drugs (cisplatin [CIS] and doxorubicin [DOX]) in standardized biopsies with increasing diameter. Then, we examined the effect of drying the biopsy surface.

## Materials and methods

### Design

This is a pharmacological experimental study in an *ex-vivo* model, examining the concentration of two chemotherapeutic drugs (CIS and DOX) in peritoneal tissue biopsies depending on pre-analytical aspects. In the first experiment, we compared the influence of the sample geometry by measuring the tissue drug concentration of punch biopsies of increasing diameter. In the second experiment, we compared drug tissue concentration with or without dabbing the superficial fluid layer on the exposed peritoneal surface.

### Sample size

We calculated the sample size needed based on pilot data (d/S) generated by Sautkin et al. [12]. Assuming an Alpha error of 0.05 and a power of 0.8, at least six probes/group are needed to reach statistical significance. We took nine biopsies from each organ to increase the confidence in our results.

### Ethical and regulatory background

No human material or living animals were employed for this study. The fresh bladders were purchased from a slaughterhouse, where the animals were bred for alimentary purposes. Thus, under German law, no authorization of the Institutional Review Board or the Animal Protection Committee was required.

### Occupational health and safety

The tested drugs are chemotherapeutics, which have a high toxicity potential and could be harmful to involved people. The two cytotoxic drugs used in the experiment are Cisplatin Teva^®^ (Teva, Ulm, Germany) and Doxorubicin HCl Teva^®^ (Teva, Ulm, Germany). The safety of NCPP laboratories was audited successfully in fall 2016. People involved received safety training. Experiments were performed in a class-3 safety hood (Maxisafe 2000, ThermoFisher Scientific, Dreieich, Germany) certified to manipulate chemotherapeutic drugs. Potential contamination of the air and surfaces was monitored by an external, independent institution (DEKRA, Stuttgart, Germany).

### *Ex-vivo* model

The enhanced Inverted Bovine Urinary Bladder (eIBUB) model has been described elsewhere [12]. Shortly, the bovine urinary bladder is an intraperitoneal organ covered mainly by peritoneum. After inversion, the bladder offers a volume similar to the human abdomen, with its inner surface lined with peritoneum. Fresh bladders were delivered on ice right before the beginning of the experiments. Each bladder was dissected, cleaned up and inverted (inside-out). A 12 mm balloon trocar (Kii^®^, Applied Medial, Düsseldorf, Germany) was introduced through the bladder neck and secured with a tight Mersilene^®^ suture. A silicone tube was sewed on the bladder bottom to evacuate in real-time the liquid dripping down. The eIBUB was inflated with CO_2_ at a pressure of 12–15 mmHg. A solution containing 2.7 mg DOX in 50 mL NaCl 0.9% and 13.5 mg CIS in 150 mL NaCl 0.9% was aerosolized at the temperature of 20–24 °C. After 30 min exposition, the toxic aerosol was safely released, and the eIBUB opened.

### Biopsies

In the first experiment, three punch biopsies of increasing diameter (6 and 12 mm) were taken at the top, middle, and bottom of the eIBUB, resulting in 3 × 3 × 2 = 18 biopsies/bladder. The biopsies were sampled perpendicular to the surface. This process was repeated in three eIBUB, resulting in a total of 27 biopsies/group, in total 54 biopsies.

**Figure 1:**
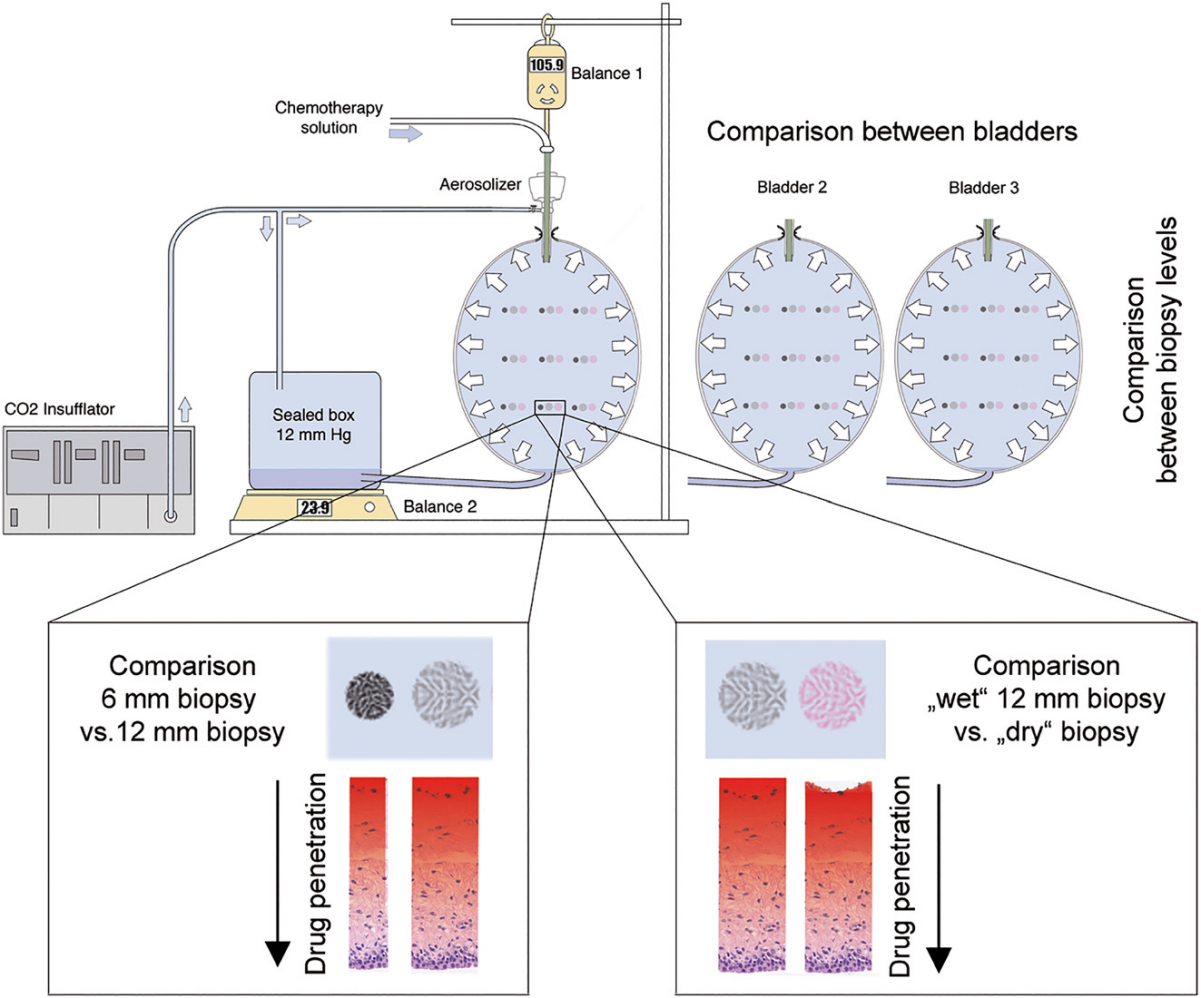
Study flow. Schematic representation of the eIBUB model and study design. Influence of biopsy geometry: 6 and 12 mm biopsies were taken at the top, middle, and bottom of the eIBUB (left box). Influence of the liquid layer on the surface: 12 mm punch biopsies were sampled at the top, middle, and bottom of the eIBUB, before and after dabbing the model's surface (right box). CIS and DOX tissue concentration was measured and compared in the different groups. All experiments were repeated in triplicate.

In the second experiment, two 12 mm punch biopsies were sampled at nine different localizations (3× top, 3× middle, and 3× bottom) of three eIBUB, resulting in 54 biopsies. The surface of every second biopsy was dabbed with soft tissue, resulting in 27 “wet” and 27 “dry” biopsies (Figure 1: Study flow)*.*

To reduce analytical costs, we used the 12 mm, wet biopsies for both experiments. All biopsies were immediately frozen and stored at −80 °C.

### Sample preparation

Sample preparation has been described elsewhere [13]. Shortly, biopsies were allowed to thaw at room temperature (RT) under a cytostatic hood. The biopsies were transferred into labeled 2 mL vials and kept at +4 °C in a fridge before lyophilization. Then, vials were placed into a Speedvac device (S-Concentrator, BA-VC-300H; H. Saur, Laborbe-darf, Reutlingen, Germany) and centrifuged under vacuum overnight (1,000 rpm; 100 mbar) at RT. The dry pellets were weighed on a high accuracy scale (R180D; Sartorius, Germany) for later normalization. Then, the dry pellets were rehydrated with 1.5 mL of sterile distilled water (Ampuwa, Fresenius KABI, Homburg, Germany) and homogenized using a homogenizer (TissueLyser LT; QIAGEN GmbH, Hilden, Germany). Shortly after, the sample material and ceramic beads were placed together into 2 mL ceramic tubes (Ceramic Bead Tubes Kit; QIAGEN GmbH, Hilden, Germany) and shaken in a vertical position (50 Hz, 3,000 oscillations/min) for 1 h at RT. Then, the tubes were placed into an ultrasounication device (Elmasonic S30H; Singen, Germany) for 10 min at RT. Finally, the tubes were mixed on a vortex mixer for 30 s, centrifuged for 10 min (5417R, 9,000 rpm; Eppendorf, Hamburg, Germany) at RT and stored at −80 °C.

### Drug concentration measurements

The deep frozen probes were sent for the analyses to a GLP certified laboratory (MVZ Dr. Eberhard & Partner Dortmund (ÜBAG), Dortmund, Germany). The laboratory worked in blind for the variant “sample identity”. The tissue concentration of DOX was measured by high performance liquid chromatography (HPLC; Waters Fluorescence Detector 2475, Waters Inc., Milford, MA), with a serum lower level of quantification (LLoQ) of 5 ng/mL. Pre-analytical validation proved a linear range of measurements in 5% glucose matrix between 0.1 and 10,000 μg/mL DOX and established no influence of organic matrices. The CIS concentration was quantified by atomic absorption spectroscopy (AAS; ZEEnit P 650, Analytic Jena AG, Jena, Germany). Pre-analytical validation proved a linear range of measurements in 5% glucose matrix between 0.1 and 100 μg/mL platinum, and established no influence of organic matrices.

### Statistical analyses

Descriptive statistics for quantitative data are given as mean and confidence intervals 5–95% and represented graphically with boxplots. Statistical comparisons between groups were performed using non-parametric tests (Mann–Whitney U-test) using SPSS software v. 25 (IBM, Chicago, USA).

## Results

**Figure 2:**
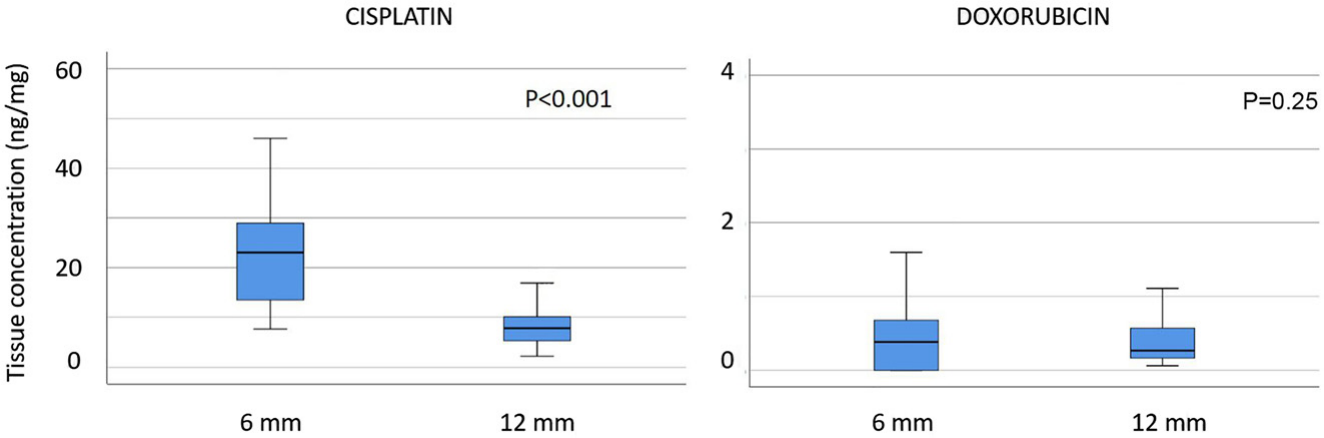
Influence of the probe geometry on tissue drug concentration. Tissue concentration of cisplatin (left panel) and doxorubicin (right panel), depending on the biopsy size. The biopsy geometry influences the measurement of cisplatin (p<0.001) but not doxorubicin (p=0.248). Thus, results of drug tissue concentration in peritoneal tissue depend on the biopsy geometry, but also the penetration of a specific drug into the tissue.

In a first experiment, we measured the tissue concentration of two different chemotherapeutic drugs (CIS and DOX) in standardized biopsies with a diameter of 6 and 12 mm, representing a surface of 28.2 versus 113.0 mm^2^, or a surface/depth ratio differing by a factor 4 between biopsies. The results are shown in Figure 2.

After normalization of the biopsy weight, the mean CIS tissue concentration was found to be significantly higher (23.2 ng/mg, CI 5–95%: 20.3–26.1) in the small biopsies than in the larger biopsies (8.1 ng/mg, CI 5–95%: 7.2–9.2), p<0.001. Concerning DOX, tissue concentration was 0.46 ng/mg (CI 5–95% 0.29–0.62) in the small biopsies vs. 0.43 ng/mg (0.33–0.54) in the larger biopsies (p=0.248). These results appeared surprising but they were reproducible between the different locations within the IBUB and also between experiments (Supplemental material, Figures 1 and 2). Thus, the geometry of the biopsy had an influence on tissue CIS concentration measurements but not on DOX.

**Figure 3:**
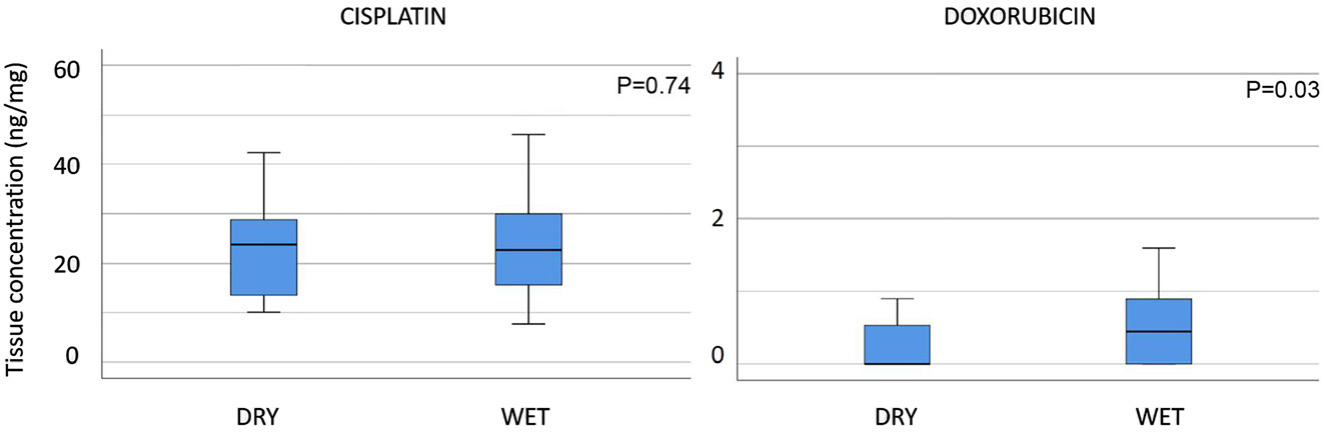
Influence of the pre-analytical probe processing on tissue drug concentration. Tissue concentration of cisplatin (left panel) does not depend on prior dabbing of the surface. In contrast, this manoeuvre influences doxorubicin concentration (right panel).

In a second experiment, we examined the effect of drying the biopsy surface on tissue drug concentration. Our hypothesis was that tissue concentration would be higher without dabbing the surface of the probe. The results are shown in Figure 3.

After normalization of the biopsy weight, the mean CIS tissue concentration did not differ between “dry” and “wet” biopsies, with respectively 23.5 (CI 5–95%: 19.0–28.0) and 22.9 (18.9–26.9) ng/mg tissue respectively (p=0.735). Concerning DOX, a statistical difference in tissue concentration was observed with 0.28 (0.12–0.43) vs. 0.64 (0.35–0.93) ng/mg tissue (p=0.025). Thus, dabbing the surface of the biopsies did meaningfully change the results of DOX tissue concentration but not CIS.

## Discussion

Many studies have been published on tissue drug uptake after intraperitoneal drug delivery, in particular after HIPEC (reviewed in [14]) but also after PIPAC (reviewed in [13]). As a rule, these studies show a significant variability of results between biopsies. To our knowledge, there was no research on the influence of the biopsy morphology and the pre-analytical steps on pharmacological measurements.

In the present research, we evaluated the tissue concentration of two cytostatic drugs commonly used for intraperitoneal chemotherapy, CIS, and DOX [15]. Cisplatin is an intercalating agent with a molecular weight of 300 kDa, an area under the curve (AUC) ratio between the peritoneal and the vascular compartments of 12–22, and an excellent depth of tissue penetration of 1,000–5,000 µm. Doxorubicin is an anthracycline with a molecular weight of 580 kDa, an AUC of 162–320, and limited tissue penetration (4–6 cell layers, representing less than 50 µm). We expected our choice of these agents, with different pharmacological characteristics, to highlight the influence of pre-analytical sample preparation on drug tissue concentration and give clues on possible differences depending on the drug used.

For this research, we used an established *ex-vivo* model of the peritoneal cavity [11, 12]. In the first experiment, we examined possible differences in tissue concentration depending on the ratio between the surface exposed to the drug and the biopsy depth. We expected drug concentration to be higher in the large biopsy (12 vs. 6 mm diameter) due to a superior surface/depth ratio. For CIS, our results show the opposite, namely that the drug tissue concentration was higher in the smaller biopsies. In contrast, tissue DOX concentration did not depend on biopsy morphology. These results are not artefactual since they were reproducible between different localizations (top, middle, and bottom) within the bladders, and between the bladders examined. Thus, biopsy morphology can influence drug tissue concentration, but this influence is depending on the drug applied.

In the second experiment, we expected that dabbing the biopsy surface would result in a lower drug tissue concentration. The experiments confirmed this hypothesis for DOX but not for CIS, where no difference was found. This finding is most likely explained by the limited uptake of DOX into the (sub-)peritoneal tissue and a significant quantity of the drug remaining at the surface after 30 min exposition time. In contrast, CIS penetrated the tissue more effectively so that dabbing the biopsy’s surface after 30 min did not significantly influence the tissue concentration measurements.

Our results highlight the need for standardizing the biopsy sampling technique by utilizing, e.g., punch biopsies certified for use in dermatology. The validity of pharmacological measurements from peritoneal biopsies taken with forceps should be questioned. Moreover, biopsies should be oriented with the peritoneum at the surface to evaluate the depth of drug tissue penetration.

In the present study, samples were collected before and after removing the superficial liquid layer before analysis, permitting an evaluation of the actual amount of drug present in the tissue. The results highlight that the fluid remaining on the peritoneal surface can influence drug concentration calculation, depending on the drug delivered.

For an anticancer drug to be effective, it has to reach a cytotoxic concentration in the whole tissue at risk. Cancer cells not reached by the drug will not be treated and will give rise to a recurrence. It is not sufficient to apply a high drug dose into the peritoneal cavity if it is not taken up effectively into tumor nodes. Our results show that pharmacological studies in peritoneal tissue have to consider different elements of the relation between drug and tissue, such as the biopsy’s morphology and a drug film remaining at the peritoneal surface. Other factors might also play a role, such as the time-gap between test-sampling and analysis, as well as the tissular drug metabolization. Previous studies in animals did not consider these standardization aspects properly and delivered measurements with high variability. Such blur prevents detection of smaller differences between delivery techniques or between different biopsies localizations within the peritoneal cavity.

In conclusion, we highlighted the influence of pre-analytical steps on drug concentration measurements in peritoneal tissue and recommend to standardize these steps, e.g. by using punch biopsies and pre-analytical surface dabbing. The implications of this simple methodological study appear significant for future research in the field.
