# Supplementary material for: Influence of pre-analytical sample preparation on drug concentration measurements in peritoneal tissue: an ex-vivo study
Source: Pleura Peritoneum. 2021 Jul 28;6(3):131–6. doi: 10.1515/pp-2020-0151 (PMC8482446; doi:10.1515/pp-2020-0151)

Supplemental Figure 1: Reproducibility between experiments for CIS and DOX.


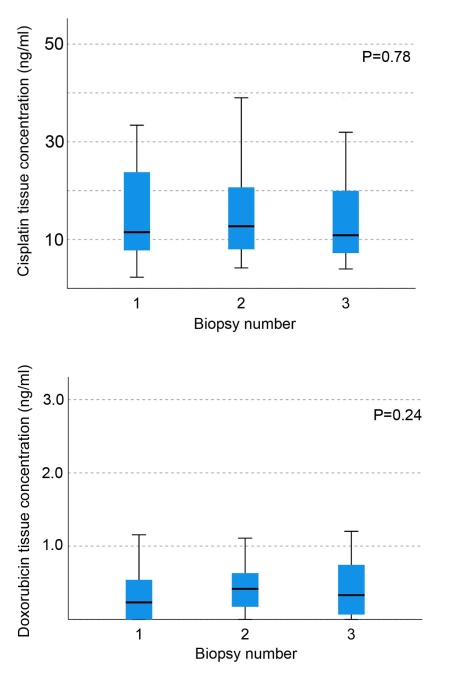


Supplemental Figure 2: Results reproducibility between the three locations on the eIBUB model (top, middle and bottom) for cisplatin and doxorubicin.


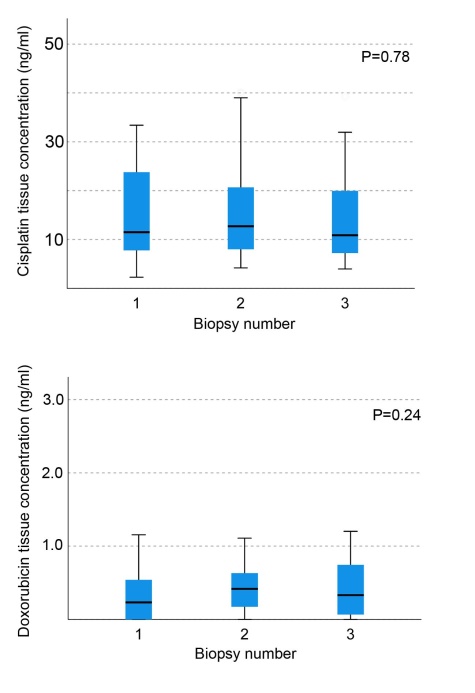

Supplement: Supplementary file 1 [file pp-06-20200151-s001.docx]
